# Patients’ Voices on Ketamine for Treatment-Resistant Depression: A Narrative Review of Qualitative Perspectives

**DOI:** 10.3390/jcm15010150

**Published:** 2025-12-25

**Authors:** Michał Walaszek, Wiesław Jerzy Cubała, Zofia Kachlik

**Affiliations:** Department of Psychiatry, Faculty of Medicine, Medical University of Gdansk, ul. Smoluchowskiego 17, 80-214 Gdańsk, Poland; wieslaw.j.cubala@gumed.edu.pl (W.J.C.); zosiakachlik@gumed.edu.pl (Z.K.)

**Keywords:** treatment-resistant depression, ketamine, patient perspectives, qualitative research, patient-reported outcomes, affective disorders

## Abstract

Treatment-resistant depression (TRD) remains a significant public-health challenge, with many patients failing to respond to conventional therapies. Ketamine has emerged as a rapid-acting intervention, but quantitative outcomes alone do not capture patients’ lived experiences, which shape engagement, acceptability, and adherence. We conducted a narrative review of qualitative and mixed-methods studies to enable conceptual integration and thematic synthesis of patients’ experiences with ketamine treatment for depression, guided by established narrative review methodology and the SANRA framework. A targeted search of MEDLINE and Scopus (November 2025) identified studies reporting adult patients’ perspectives on therapeutic ketamine or esketamine use, with qualitative data synthesized iteratively in keeping with narrative review principles. Across the literature, patients’ perspectives coalesce around key thematic domains, including motivations and expectations for treatment, the phenomenology of the treatment experience, post-treatment trajectories, side effects and reasons for discontinuation, relational and environmental factors, and information and education needs. By focusing on these thematic groups, the review highlights the experiential dimensions that influence the perceived value and acceptability of ketamine, underscoring the need for patient-centered service design. Integrating these insights can guide the development of ketamine programs that are both evidence-based and aligned with patients’ priorities and perspectives.

## 1. Introduction

Major depressive disorder (MDD) is a leading cause of disability worldwide and a major contributor to global disease burden, associated with substantial personal suffering, impaired functioning, increased mortality, and significant societal and economic costs [1]. Despite the availability of multiple pharmacological and psychotherapeutic interventions, a considerable proportion of individuals with depression fail to achieve sustained remission, even after sequential, guideline-concordant treatments [2]. This subgroup of patients, referred to as having treatment-resistant depression (TRD), experiences a more severe and persistent course of illness, with disproportionately high risks of suicidality, functional disability, and health-care utilization [3].

In recent years, ketamine has emerged as a rapid-acting intervention capable of producing antidepressant effects within hours and sustaining improvement for several days [4]. Randomized controlled trials and large-scale real-world cohorts have consistently demonstrated its short-term efficacy and acceptable safety profile in TRD [5,6]. Nevertheless, quantitative outcomes alone cannot capture how patients experience and interpret ketamine treatment, nor how these experiences shape engagement, perceived legitimacy, and long-term acceptability. Qualitative research offers a necessary complement to efficacy-focused approaches by illuminating patients’ lived experiences of treatment. Evidence from qualitative studies across mental health interventions indicates that misalignment between clinician-defined outcomes and patient-valued experiences can undermine treatment engagement, distort risk–benefit appraisals, and contribute to service designs that fail to meet patients’ needs [7]. In the context of ketamine, an intervention involving altered states of consciousness, off-label use, and ongoing ethical debate, patients’ subjective perspectives are particularly consequential for informed consent, shared decision-making, and responsible clinical implementation.

Despite a growing number of qualitative studies examining patient experiences with ketamine, this area of research remains fragmented and has not been comprehensively synthesized. Many studies are small, cross-sectional, and heterogeneous in focus, limiting their capacity to inform patient-centered care at a system level. Given the rapid expansion of ketamine use in TRD and the accumulation of qualitative research published in recent years, a narrative synthesis of patients’ perspectives is both timely and necessary.

Accordingly, this narrative review focuses on TRD and synthesizes qualitative and mixed-methods studies that explicitly report patients’ lived experiences of ketamine or esketamine treatment. All clinically administered routes of administration are considered, including intravenous, intranasal, oral, and other formulations. The review does not aim to evaluate quantitative efficacy or safety outcomes. Instead, it seeks to (i) delineate the thematic landscape of patients’ subjective experiences with ketamine for TRD; (ii) examine practical and experiential factors influencing treatment acceptability and accessibility; and (iii) identify patient-centered priorities for future research and clinical implementation.

## 2. Methods

A narrative approach was selected to facilitate conceptual integration, thematic synthesis, and critical interpretation of diverse qualitative findings, in line with established guidance for narrative reviews [8]. The conduct and reporting of the review were informed by the SANRA (Scale for the Assessment of Narrative Review Articles) framework to support clarity, coherence, and methodological transparency [9].

A targeted literature search was performed in MEDLINE (via PubMed) and Scopus in November 2025 using the following terms: (ketamine OR esketamine) AND (depression OR “treatment resistant depression” OR TRD) AND (qualitative OR interview OR “focus group” OR “patient experience” OR “lived experience” OR perspective). Eligible studies included those involving adults with depression, including TRD, that examined ketamine or esketamine administered for antidepressant treatment and reported qualitative data derived from interviews, focus groups, open-ended survey responses, or qualitative components of mixed-methods designs, with explicit reporting of patients’ perspectives. Studies were excluded if they focused solely on quantitative symptom outcomes, presented clinician perspectives without patient-reported data, or addressed recreational or non-therapeutic ketamine use. Study selection was conducted iteratively, consistent with narrative review methodology, with inclusion criteria refined as engagement with the literature progressed.

## 3. Patients’ Lived Experiences with Ketamine for Treatment-Resistant Depression

**Figure 1:**
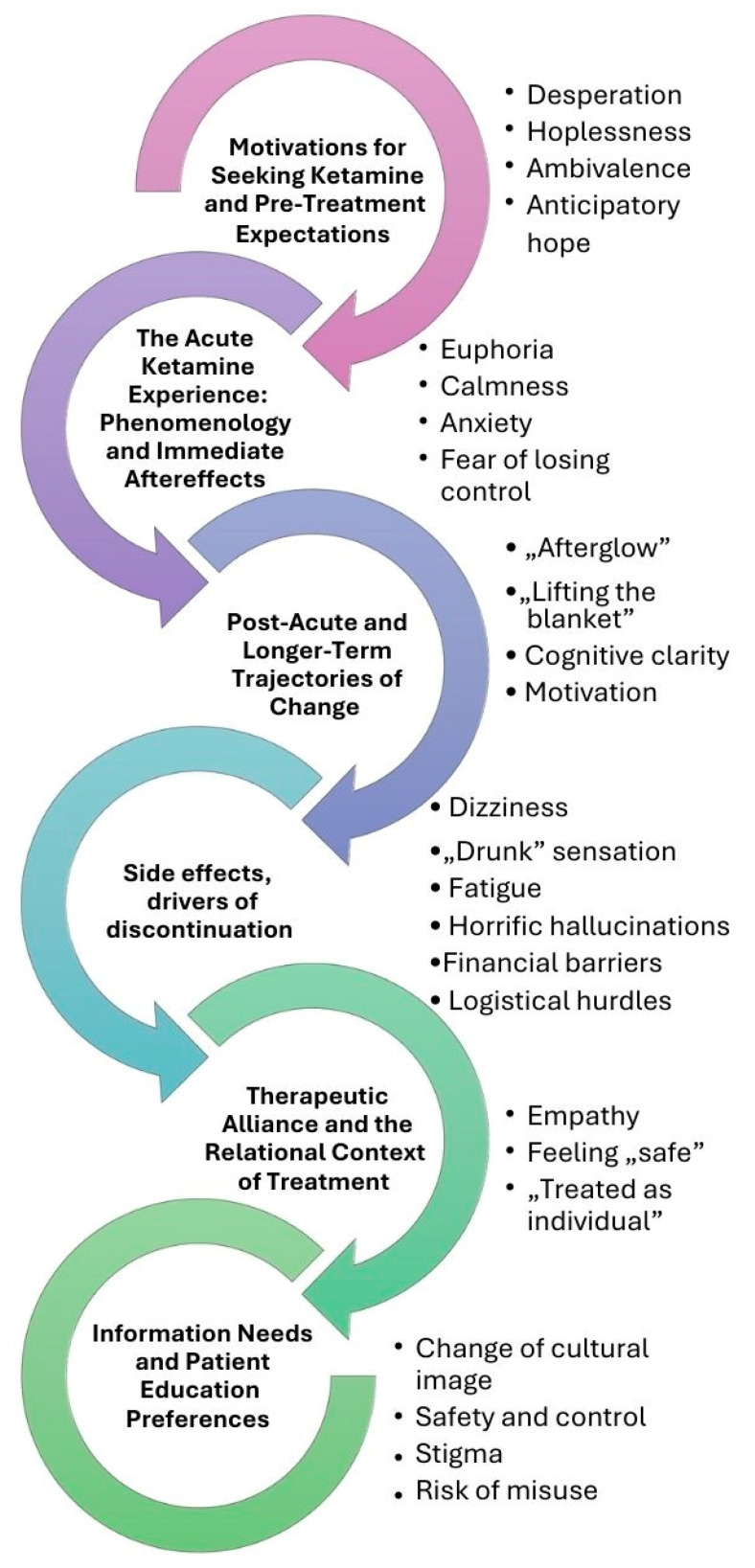
Chronological, patient-centered trajectory of ketamine treatment with key themes across phases.

Table 1 represents the thematic framework of included original studies. Figure 1 presents a conceptual, patient-centered synthesis of ketamine treatment, illustrating how patients’ motivations, acute experiences, longer-term meanings, and reasons for continuation or discontinuation unfold over time. The figure is intended to guide the thematic narrative by situating individual themes within a chronological treatment trajectory.

### 3.1. Motivations for Seeking Ketamine and Pre-Treatment Expectations

Ketamine is not a first-line intervention for depression; instead, it is introduced later in the treatment pathway, typically as an adjunct to antidepressants in cases of treatment resistance [18]. Entering ketamine treatment therefore occurs within a context marked by repeated therapeutic failures, and this history strongly shapes patients’ emotional responses (Figure 1). Many describe a mounting sense of desperation after numerous unsuccessful interventions and prolonged, severe depressive episodes. Qualitative studies consistently report individuals feeling they had “run out of options” and were willing to try “almost anything” to regain basic functioning, positioning ketamine as a last-resort attempt to initiate any form of recovery [10].

At the same time, this desperation often coexists with a cautious form of anticipatory hope. For some patients, ketamine represents the possibility, sometimes the first in years, of meaningful mood improvement or even the chance to rebuild aspects of life lost to TRD [11]. For others, however, the memory of repeated failures tempers this optimism. Prior disappointments generate ambivalence or persistent hopelessness regarding whether ketamine will truly differ from previous treatments [11].

### 3.2. The Acute Ketamine Experience: Phenomenology and Immediate Aftereffects

Across intravenous and oral studies, patients report a rapid departure from ordinary consciousness marked by perceptual distortions of body, space and time (e.g., “my hands felt backwards,” “I was floating in an empty void”). Visual and auditory changes range from colorful spirals and geometric patterns to amplified ambient sounds, often described as “dream-like” or “pseudo-hallucinatory” because existing sensory input is warped rather than replaced [12]. A pronounced “high” and heightened sense of openness coexist with feelings of detachment from self, emotions, and the external world [13,14]. Affective tone is highly variable: many experience an initial surge of euphoria, calmness and lightness, while others encounter anxiety, fear of losing control, or panic when the dissociative intensity exceeds expectations [12,14]. These affective swings are typically experienced as a rapid “roller-coaster” that subsides into a gentle comedown [12,14]. Notably, studies using different ketamine enantiomers confirm these features-perceptual changes, detachment, and mystical-type effects are characteristic of ketamine regardless of the route of administration, though psychological distress remains a common element [13]. The subjective quality of the experience is further shaped by context; patients emphasized that the “set” (mindset and expectations) and the “setting” (a quiet, professional environment) heavily influenced whether the acute effects were perceived as therapeutic or distressing [14].

### 3.3. Post-Acute and Longer-Term Trajectories of Change

Beyond the acute dissociative state, patients frequently describe a distinct post-acute “afterglow” in the hours and days following ketamine (Figure 1). Mood often shifts from oppressive negativity to a lighter, more neutral or positive tone, sometimes captured as “lifting the blanket”, with worries feeling less emotionally charged and more like concrete tasks that can be approached and solved [13,19]. Rather than a simple on/off antidepressant effect, this phase is experienced as a graded hedonic shift: increased energy, motivation and cognitive clarity alongside reduced rumination, anxiety and suicidal ideation, typically lasting from several days up to 2–3 weeks in many reports [10,11,12,15]. Patients commonly note that relationships feel easier, they feel closer to others, and everyday activities become achievable again, echoing themes of renewed agency and personal growth [10,19]. For a subset, these shifts consolidate into more chronic changes in functioning, quality of life and social connectedness, especially when treatment is embedded within ongoing care and psychotherapy [15,16]. However, many experience a gradual erosion of benefits, with mood and suicidality returning toward baseline over weeks, which can provoke frustration, grief and renewed hopelessness [11,19].

### 3.4. Side Effects and Non-Financial Drivers of Discontinuation

Across studies, most patients report side-effects during or shortly after ketamine that are transient but sometimes intense (Figure 1). Common physiological effects include dissociation, dizziness, “heavy” or “drunk” sensations, blurred vision, nausea, headaches, fatigue and short-lived blood-pressure increases [11,16]. Dissociative and perceptual changes are almost universal and can range from mildly “spacey” or dream-like states to profound out-of-body experiences, ego dissolution and near-death-type phenomena [10,19]. Emotionally, sessions may contain waves of euphoria, calm and laughter but also anxiety, panic, paranoia, grief and resurfacing of traumatic material, which some describe as overwhelming or even potentially traumatic [10,19].

In most qualitative cohorts, these effects are accepted as part of treatment and are framed by patients as tolerable, especially when they are well-prepared, monitored and supported by trusted staff [14,16]. However, a minority experience side-effects serious enough to stop or modify treatment: examples include “horrific” hallucinations, extreme panic, or a debilitating 4-day headache that led to permanent discontinuation after a single infusion [10,11]. In oral esketamine programs, frightening early sessions, inadequate preparation and feeling left alone have produced lasting aversion that colors later sessions and may contribute to dropout [14].

More often, reasons for quitting are not side-effects alone but their interaction with other disappointments: loss of initial efficacy, relapse between sessions, the emotional crash when benefits wane, cost and travel burden, and fear of dependency or future withdrawal of treatment [11,20]. When ketamine fails after high hopes, some patients describe feeling “worse” and more hopeless, highlighting the ethical importance of honest expectation management and robust follow-up when treatment is stopped [11,19].

### 3.5. Potential of Misuse

Qualitative studies consistently indicate that concerns about the potential for ketamine misuse and addiction are salient in patients’ narratives, even in the absence of personal experience with the drug. Participants described apprehensions shaped by media portrayals and public discourse emphasizing addiction risk, which contributed to stigma surrounding ketamine treatment and influenced expectations and acceptability [21].

These patient-reported concerns coexist with findings from quantitative safety literature, which have not identified evidence of abuse or dependence in clinical trials of ketamine or esketamine for depression. A systematic review encompassing randomized controlled trials, open-label studies, retrospective chart reviews, and case reports of oral ketamine reported no cases of abuse or dependence, and similarly, randomized trials of intranasal esketamine observed no instances of dependence, craving, or addiction during treatment periods of up to 28 days [22,23]. This apparent divergence reflects differences between population-level clinical evidence and individual meaning-making shaped by media narratives and social context, rather than a direct contradiction. While such data provide important clinical context, the persistence of addiction-related fears in qualitative accounts underscores the role of social narratives and stigma in shaping patients’ experiences.

### 3.6. Barriers and Accessibility

Because ketamine’s patent has expired, there is little commercial incentive for manufacturers to pursue broader reimbursement, leaving most clinics to operate on a fee-for-service model; participants therefore rely on personal funds or limited private-clinic subsidies, which many consider inadequate [24]. All participants in interview-based study describe the out-of-pocket cost of each infusion as prohibitive, noting that high fees limit continued access, force treatment discontinuation, and contribute to feelings of hopelessness [21]. Several respondents contrasted the expense of proactive ketamine therapy with the far greater cost of hospitalization, questioning the efficiency of allocating limited financial resources to reactive care [25].

Beyond price, geographic scarcity of treatment sites compounds the expense. Several patients reported living far from the few clinics that offer supervised ketamine, requiring costly travel, time off work, and often a companion to accompany them during the mandatory monitoring period [25]. This logistical hurdle magnifies the financial strain and can lead to missed appointments or delayed care.

### 3.7. Therapeutic Alliance and the Relational Context of Treatment

The interpersonal and environmental context consistently emerges as a major determinant of how patients experience, interpret and use ketamine intervention (Figure 1). Patients describe feeling “safe,” “looked after” and “treated as an individual” when staff are caring, well-prepared and available, and many explicitly frame the therapeutic relationship and clinic atmosphere as a core part of the benefit, sometimes “almost 50%” of what ketamine offers [10]. Supportive alliances help patients tolerate intense dissociation, anxiety and resurfacing memories, transforming these into meaningful, manageable experiences rather than traumatizing events, especially when clinicians prepare them, remain present during sessions, and offer space to debrief and integrate afterwards [14]. Patients value specialist settings and empathic staff who understand severe depression, prefer ketamine to be delivered in such clinics rather than in rushed primary care, and often become active advocates for wider access when they feel held by the team [16,17]. Conversely, feeling alone, insufficiently informed or unable to contact staff amplifies fear, colors subsequent sessions and can contribute to disengagement [14,19]. When treatment does not meet high expectations, the quality of the therapeutic relationship appears to shape whether disappointment is experienced as a manageable setback or as a deepening of hopelessness, underscoring the ethical importance of expectation management and follow-up [11,19]. Qualitative syntheses therefore argue for embedding ketamine within ongoing, relational care with proactive monitoring and adjunctive psychological support, rather than as an isolated pharmacological procedure [11,14].

### 3.8. Information Needs and Patient Education Preferences

Patients emphasize that good information and public education must tackle ketamine’s cultural image as a “horse tranquilizer” and “party drug” more than technical details alone (Figure 1). Many enter treatment aware of its recreational reputation and fear being judged by family, employers, or insurers, which makes them reluctant to disclose use. They therefore want clear, accessible explanations that differentiate medically supervised, low-dose ketamine or esketamine from illicit use, highlighting differences in dose, purity, indication, and tight regulation [17]. Some suggest branding or naming strategies that distinguish clinical formulations (e.g., esketamine) from “ketamine” to signal safety and control, while others warn that renaming must not obscure the fact that it is the same drug and could undermine trust if perceived as “hiding” ketamine [17]. Patients also call for broader public-facing education campaigns to counter stigma and normalize ketamine as one option among other evidence-based treatments, rather than a last-resort or exotic intervention [17].

Within services, they prefer preparatory conversations that include realistic descriptions of altered states: dissociation, time distortion, paranoia, awe or spiritual-type experiences, framed as transient, modulated by setting, and manageable with support, rather than being reduced to the label of a “dissociative anesthetic” [14]. Some authors note that this narrow label fails to capture the full experiential range and advocate new language and measurement tools that also acknowledge potentially meaningful or transformative aspects of sessions. Patients endorse this more nuanced, person-centered framing, including use of first-person accounts from previous recipients, empathetic discussion of misuse and diversion risks, and personalized, destigmatizing explanations that help them place ketamine within their own cultural and moral worlds [14,16].

## 4. Discussion

A central methodological and conceptual challenge in ketamine research lies not merely in the choice of outcome measures, but in a broader mismatch between how treatment success is operationalized within biomedical research and how therapeutic change is experienced and understood by patients. Current frameworks privilege clinician-rated, symptom-focused, and acutely observable phenomena, whereas qualitative data consistently show that patients define benefit in relational, experiential, and meaning-based terms that unfold over time [13].

This tension is most clearly illustrated by the continued prominence of the Clinician-Administered Dissociative States Scale (CADSS) as a standard marker of acute ketamine effects. Rooted in dissociation research from PTSD, the CADSS reflects a clinician-centered epistemology in which dissociation is treated as a measurable, potentially mechanistic phenomenon. However, converging evidence now indicates that dissociation is neither a biomarker nor a prerequisite for antidepressant response [26,27]. Post hoc analyses of phase 3 esketamine trials consistently demonstrate no robust association between CADSS scores and improvements in depressive symptoms or suicidality [27,28]. Patients who experience minimal or no dissociation may still derive substantial benefit, directly contradicting implicit assumptions embedded in monitoring practices.

At the same time, qualitative studies consistently identify therapeutic experiences that fall outside the conceptual scope of dissociation altogether. Patients describe shifts in perspective, interruptions of negative cognitive patterns, feelings of unity or emotional release, and moments of insight that are often retrospectively framed as meaningful or transformative [29,30,31]. The repeated calls from qualitative researchers for new or specialized measures (e.g., Mystical Experience Questionnaire, Challenging Experiences Questionnaire, Emotional Breakthrough Inventory) highlights the need to capture the full phenomenological range of acute effects, including the potentially therapeutic or transformative dimensions [29,30,31].

However, expanding acute phenomenological measures alone is insufficient. What patients ultimately emphasize is not simply what they feel during a session, but how the treatment fits into their lives, relationships, and sense of agency. Patient-reported outcome measures such as the Treatment Satisfaction Questionnaire for Medication (TSQM-9) offer a way to quantify perceived effectiveness, convenience, and overall satisfaction—domains largely invisible to traditional clinician-centered scales [32]. These dimensions directly influence motivation, adherence, and willingness to continue treatment, particularly in interventions characterized by transient effects and high financial burden [16]. In this sense, satisfaction is not a secondary outcome but a mediator between short-term symptom change and sustained engagement.

The problem of misalignment is further compounded by temporal ambiguity. Qualitative research delineates at least three distinct phases of the ketamine experience: a pre-treatment phase often marked by desperation or “last resort” motivation; an intense acute session; and a gradual post-treatment trajectory commonly described as an “afterglow” [12]. Yet quantitative studies rarely standardize the time windows to which assessments refer, blurring distinctions between transient psychoactive effects and longer-lasting antidepressant or functional changes. As a result, phenomena such as euphoria, detachment, or cognitive clarity are inconsistently interpreted as either side effects or therapeutic outcomes. Establishing shared temporal frameworks (e.g., peri-session, 0–24 h, days 1–7, >7 days) and mapping both subjective experience and functional recovery onto these phases would represent a critical step toward conceptual coherence.

Crucially, patient accounts also challenge the implicit assumption that ketamine’s effects are purely pharmacological. Across studies, patients repeatedly identify the relational context, empathetic staff, a sense of safety, and feeling emotionally held, as integral to therapeutic benefit, sometimes attributing equal or greater importance to these elements than to the drug itself [10]. This finding complicates dominant biomedical narratives by suggesting that ketamine functions within a psychosocial container, in which relational factors shape how intense or frightening experiences are integrated into meaningful change. From this perspective, dissociation, anxiety, or perceptual alterations are not intrinsically therapeutic or harmful; their impact depends on whether they occur within a supportive, interpretive framework.

Finally, these issues intersect with broader cultural stigma surrounding ketamine. Patients are acutely aware of its association with recreational use and frequently fear judgment or moral scrutiny [17]. When combined with the high likelihood of relapse after discontinuation and the financial inaccessibility of long-term treatment, this stigma can exacerbate hopelessness and distress [11]. Failure to address these concerns systematically risks reproducing cycles of expectation inflation followed by disillusionment. Ethical clinical practice therefore requires proactive destigmatization, transparent expectation management, and structured follow-up when treatment ends.

Taken together, these findings suggest that the central challenge in ketamine therapy is not identifying a single mechanistic predictor of response, but redefining what constitutes meaningful benefit. Future research should move beyond narrowly defined symptom reduction and instead integrate standardized temporal frameworks, patient-reported satisfaction, relational context, and qualitative insight into core outcome models. Such an approach would not only improve measurement validity but also support more humane, transparent, and sustainable models of care grounded in patient-defined recovery rather than clinician-imposed proxies [14].

## 5. Limitations

Several limitations should be considered when interpreting the findings of this narrative review. First, the qualitative studies included were typically based on small and purposive samples, which is appropriate for exploratory qualitative research but limits the generalizability of the reported experiences. Participants were often recruited from specialized services or research settings and may not reflect the full range of individuals receiving ketamine treatment for treatment-resistant depression.

Second, many studies drew on samples of patients who continued treatment or agreed to participate in interviews, introducing a potential selection bias toward individuals with more acceptable or meaningful treatment experiences. Perspectives of patients who discontinued ketamine early, experienced adverse effects, or declined further treatment may therefore be underrepresented.

It is important to note that patient experience may plausibly vary by route of administration (e.g., intravenous, intranasal, oral), treatment setting, dosing schedule, and treatment phase (acute induction versus maintenance). However, the current qualitative literature rarely disaggregates themes along these dimensions, and most reported findings emerge across heterogeneous protocols. As such, themes related to therapeutic alliance, expectation management, stigma, and post-treatment meaning-making appear broadly generalizable, whereas differences in the intensity or qualitative character of acute psychoactive effects likely reflect context-specific factors that remain insufficiently studied.

Finally, limitations inherent to the narrative review methodology must be acknowledged. The synthesis was interpretive rather than systematic, and themes were developed through iterative engagement with the literature rather than formal qualitative coding or meta-synthesis. While this approach allows conceptual integration across heterogeneous studies, it may be more susceptible to subjectivity and does not aim to provide exhaustive coverage or reproducible theme generation.

## 6. Conclusions

Ketamine can deliver rapid, sometimes transformative relief for TRD, yet patients describe a wide spectrum of acute effects, after-effects, and side-effects that are strongly influenced by their expectations, the therapeutic setting, and the quality of staff support. Across qualitative studies, the therapeutic alliance, thorough preparation, integration support, and clear, destigmatizing education emerged as essential for tolerating intense experiences and sustaining benefit, while inadequate support, relapse, cost, and stigma often precipitate disengagement.

Patients’ perspectives highlight concrete service design priorities, including structured pre-treatment preparation, staff continuity during sessions, proactive follow-up when benefits wane, and explicit discussion of stigma, dependency concerns, and discontinuation. Consequently, ketamine services should be embedded in ongoing, relational care that employs patient-reported outcome and experiential measures, standardized temporal windows, and individualized counseling to align clinical practice with patients’ own definitions of safety, satisfaction, and therapeutic success.

## Figures and Tables

**Table 1:** Included qualitative studies on patient perspectives of ketamine treatment.

Study Title	Reference Number	Number of Participants	Route	Setting	Qualitative Method	Themes
A qualitative study of patients’ experience of ketamine treatment for depression: The ‘Ketamine and me’ project	[10]	13	i.v.	Outpatient clinic	Semi-structured interviews and interpretative phenomenological analysis (IPA)	Treatment decision influencers Responses experienced taking ketamine Treatment enhancers Barriers to treatment efficacy and success
Ketamine treatment for individuals with treatment-resistant depression: longitudinal qualitative interview study of patient experiences	[11]	12	i.v. and p.o.	Outpatient clinic and taken at home	Semi-structured interviews and inductive semantic approach	Pre-treatment Impact of treatment on mood and suicidal ideation, Initial impact (first two treatments) Subsequent impact (third treatment onward) Side-effects of ketamine treatment Overall perspectives on treatment
A qualitative and quantitative account of patient’s experiences of ketamine and its antidepressant properties	[12]	32	i.v.	Inpatient unit	Semi-structured interviews and reflexive thematic analysis approach	**Acute:** Change in perception Emotional or mood changes Loss of control Questioning of existence or self Physiological **Final**: Change in perspective Change in mood Change in emotion Time Expectations Future treatments
Phenomenology and therapeutic potential of patient experiences during oral esketamine treatment for treatment-resistant depression: an interpretative phenomenological study	[13]	17	p.o.	Inpatient unit	In-depth interviews, conducted within an Interpretative Phenomenological Analysis (IPA) framework	**Acute (during session) experiences:** Perceptual effects Detachment Tranquility and openness Mystical-type experiences Fear and anxiety Variability **Post-session experiences:** Feeling hungover and fatigued Lifting the blanket: mood effects
Holding on or letting go? Patient experiences of control, context, and care in oral esketamine treatment for treatment-resistant depression: A qualitative study	[14]	17	p.o.	Inpatient unit	In-depth interviews, conducted within an Interpretative Phenomenological Analysis (IPA) framework	Overwhelming experiences Inadequate preparation Side effects or core treatment components? Hope and expectations Letting go of control Mood states influencing session experience Presence and emotional support Supportive presence Emotional support and trust Support with integration Supportive settings (Lack of) privacy and silence A warm, comfortable environment Rituals and strategies to optimize effects The role of music
A Quantitative and Qualitative Analysis of the Patient and Caregiver’s Perspective on Outcomes of Intravenous Administration of Low-Dose Ketamine for C-PTSD, TBI, and Treatment Resistant MDD: A Clinical Example	[15]	1	i.v.	Outpatient clinic	Clinical interview	NA (Case report)
Patient Experience with Intranasal Esketamine in Treatment-Resistant Depression: Insights from a Multicentric Italian Study (REAL-ESKperience)	[16]	236	i.n.	Outpatient clinic	Open-field responses thematically clustered via ChatGPT-4	Personal well-being Subjective experience Environment, and protocol setting
Ketamine treatment for depression: qualitative study exploring patient views	[17]	14	NA	Lack of specific description	Focus groups and thematic analysis by inductive method	Changing public perceptions of ketamine Risk related to procurement, side-effects and use of ketamine Monitoring ketamine use Privacy and data protection around ketamine treatment Practical aspects of ketamine use

i.v.—intravenous; p.o.—per os; i.n.—intra nasal; NA—not available/applicable.

## Data Availability

No new data were created or analyzed in this study.

## Abbreviations

The following abbreviations are used in this manuscript:

TRD	Treatment-resistant depression
CADSS	Clinician-Administered Dissociative States Scale
TSQM-9	Treatment Satisfaction Questionnaire for Medication
